# Whole-genome analysis of *Fusarium graminearum* insertional mutants identifies virulence associated genes and unmasks untagged chromosomal deletions

**DOI:** 10.1186/s12864-015-1412-9

**Published:** 2015-04-03

**Authors:** Martin Urban, Robert King, Keywan Hassani-Pak, Kim E Hammond-Kosack

**Affiliations:** Department of Plant Biology and Crop Science, Rothamsted Research, Harpenden, Herts AL5 2JQ UK; Department of Computational and Systems Biology, Rothamsted Research, Harpenden, Herts AL5 2JQ UK

**Keywords:** Virulence mutant analysis, *Gibberella zeae*, Forward genetics, Next generation sequencing, Filamentous plant pathogen

## Abstract

**Background:**

Identifying pathogen virulence genes required to cause disease is crucial to understand the mechanisms underlying the pathogenic process. Plasmid insertion mutagenesis of fungal protoplasts is frequently used for this purpose in filamentous ascomycetes. Post transformation, the mutant population is screened for loss of virulence to a specific plant or animal host. Identifying the insertion event has previously met with varying degrees of success, from a cleanly disrupted gene with minimal deletion of nucleotides at the insertion point to multiple-copy insertion events and large deletions of chromosomal regions. Currently, extensive mutant collections exist in laboratories globally where it was hitherto impossible to identify all the affected genes.

**Results:**

We used a whole-genome sequencing (WGS) approach using Illumina HiSeq 2000 technology to investigate DNA tag insertion points and chromosomal deletion events in mutagenised, reduced virulence *F. graminearum* isolates identified in disease tests on wheat (*Triticum aestivum*). We developed the FindInsertSeq workflow to localise the DNA tag insertions to the nucleotide level. The workflow was tested using four mutants showing evidence of single and multi-copy insertions in DNA blot analysis. FindInsertSeq was able to identify both single and multi-copy concatenation insertion sites. By comparing sequencing coverage, unexpected molecular recombination events such as large tagged and untagged chromosomal deletions, and DNA amplification were observed in three of the analysed mutants. A random data sampling approach revealed the minimum genome coverage required to survey the *F. graminearum* genome for alterations.

**Conclusions:**

This study demonstrates that whole-genome re-sequencing to 22x fold genome coverage is an efficient tool to characterise single and multi-copy insertion mutants in the filamentous ascomycete *Fusarium graminearum.* In some cases insertion events are accompanied with large untagged chromosomal deletions while in other cases a straight-forward insertion event could be confirmed. The FindInsertSeq analysis workflow presented in this study enables researchers to efficiently characterise insertion and deletion mutants.

**Electronic supplementary material:**

The online version of this article (doi:10.1186/s12864-015-1412-9) contains supplementary material, which is available to authorized users.

## Background

Insertional mutagenesis is a widely used and powerful technique to discover and study the function of genes in most organisms. In pathogenic microbial organisms mutagenesis has played a pivotal role in early gene discovery and is still used alongside more targeted gene deletion approaches for gene function analysis.

In pathogenic fungi the methods applied include random marker gene insertion using plasmids [1], *Agrobacterium tumefaciens* transformation (ATMT) [2] and transposon mutagenesis [3]. These forward genetic methods aim to be relatively unbiased, but hotspots of insertion events are sometimes reported [3]. Mutants are generated and tagged by nucleotide changes or by the integration of a transgenic marker gene into critical promoter regions or into protein encoding sequences. The affected genes frequently show altered gene expression and/or the generation of truncated proteins. In mutant screens, typically hundreds or thousands of mutants are generated and screened for a desired alteration in a specific phenotype. For filamentous haploid plant pathogens with moderate sized genomes typically the loss of pathogenicity is tested in glasshouse or controlled environment based screens using the most suitable host plant and bioassay. Once relevant mutants are identified, tagged gene(s) are isolated using a variety of methods. These methods include the use of genetic maps and genome walking [4], polymerase chain reaction (PCR) based approaches [2] or plasmid rescue [1].

More recently the introduction of affordable high-throughput sequencing techniques has facilitated the analysis of whole genomes [5]. These methods include the currently widely used Illumina sequencing-by-synthesis approach with fluorescently labelled reversible-terminator nucleotides [6]. The availability of reference genomic information for many species allows the characterisation of mutants using whole-genome sequencing (WGS). Such an approach was earlier reported for bacterial and protozoa species of small genome sizes up to 23 MB to identify transposon insertion mutations [7,8]. Also for two saprophytic ascomycete model fungi of 40 Mb genome size, WGS was shown to be suitable for the identification of developmental genes and narrowing down of candidate mutations induced by chemical, UV and γ-radiation [9-11]. However for pathogenic filamentous fungi this technology is not successfully established. Previous WGS approaches have been hindered by a combination of factors. These factors include, poorly designed methodologies, suboptimal sequence read length, the library size used during sequencing and the presence of high amounts of repetitive DNA in a range of microbial pathogens such as *Magnaporthe oryzae* (10%).

*Fusarium graminearum* (teleomorph *Gibberella zeae*) is a globally important pathogen of wheat, maize and barley. The pathogen belongs to the filamentous ascomycetes and has a genome of 37 Mb in length, distributed over predominantly four chromosomes. A high quality genome assembly exists and this is aligned to a genetic map [12,13]. *F. graminearum* is a haploid organism and effective transformation techniques are established [14]. A natural gene silencing mechanism called Repeat induced point (RIP) mutation exists in *F. graminearum* which mutates duplicated sequences [12]. Hence inspection of the genomic DNA sequence revealed the presence of only a minimal amount of repetitive DNA. *F. graminearum* therefore appears to be ideally suited to test a whole-genome sequencing approach for insertion mutant characterisation.

In this study we explored a whole-genome sequencing approach using the Illumina HiSeq 2000 platform to identify insertion points and possible genomic rearrangements in four selected plasmid insertion mutants with reduced virulence to wheat*.* The analysed mutants included low, medium and high-complexity insertion events, pre-screened using Southern (DNA gel blot) analysis. We set up a *de novo* bioinformatics workflow called FindInsertSeq. The FindInsertSeq bioinformatic pipeline successfully identified DNA tag insertion sites with a precision to the nucleotide level for the four mutants analysed. Subsequent sequenced read coverage inter-comparisons of the mutant genomes revealed additional recombination events including larger tagged and untagged chromosome deletions, and DNA amplification events. Using a random data sampling approach, the minimal genome coverage required for the FindInsertSeq workflow to provide reliable results was determined to be 22× fold. We propose that WGS and the FindInsertSeq approach is an effective method to characterise insertional mutants and transgenic strains expressing constructs of interest in filamentous fungi.

## Results

### Isolation of reduced virulence *F. graminearum* mutants with insertion events of different complexities

**Figure 1:**
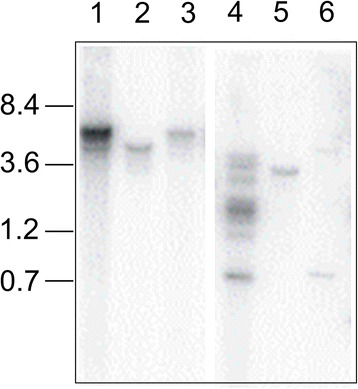
**DNA blot analysis of three mutants showing low, medium and complex insertion events.** DAF mutant genomic DNA was digested with two restriction enzymes and probed with a radioactive labelled *hph* gene. Lanes 1 to 3: *EcoRI* digested DNA of DAF139, DAF140 and DAF141. Lanes 4 to 6: *XhoI* digested DNA of DAF139, DAF140, DAF141.

An insertional mutagenesis screen of the *F. graminearum* wild-type strain PH-1 was completed using a linearised plasmid vector containing the hygromycin phosphotransferase gene under control of the *trpC* promoter. In total, 650 stable transformants were generated and screened on wheat ears of the cultivar Bobwhite for reduced virulence using previously described methods [15]. Eight mutants that showed highly reduced virulence were identified (full details on their phenotype characterisation will be published elsewhere). Insertion events were characterised using Southern blot analysis and three of the Disease Attenuated Fusarium mutants, called DAF139, DAF140 and DAF141 were chosen for analysis in this study (Figure 1). A fragment of the *hph* phophotransferase resistance gene was used as a probe. The mutagenesis plasmid pHYG1.4 has single restriction sites for *EcoRI* and *XhoI* (Additional file 1: Figure S1). For DAF140, genomic DNA was cut with both enzymes to produce single hybridisation bands indicating a single insertion event. For DAF141 the *XhoI* digest revealed two fragments at 0.8 and 5 kb consistent with the insertion of more than one possibly rearranged plasmid. For DAF139 the presence of multiple hybridisation fragments of different sizes indicated a highly complex insertion event. The fourth mutant explored as a control in this study was the previously characterised non-virulent *F. graminearum* strain TP11.1. This strain was previously demonstrated in a targeted PCR based analysis to harbour a single insertion event in the *TOP1* gene (FGSG_06874) and was generated using the same plasmid [15].

### Sequencing and FindInsertSeq bioinformatics approach to identify insertion tags and transformation-induced mutations

Illumina sequencing was used to obtain high whole-genome coverage for the three DAF mutants and the control strain TP11.1. Genome coverage of 96 to 110 times was achieved by 100-bp paired-end reads from inserts with a mean library fragment size of 430 bp on an Illumina HiSeq 2000 system (Additional file 1: Table S1).

**Figure 2:**
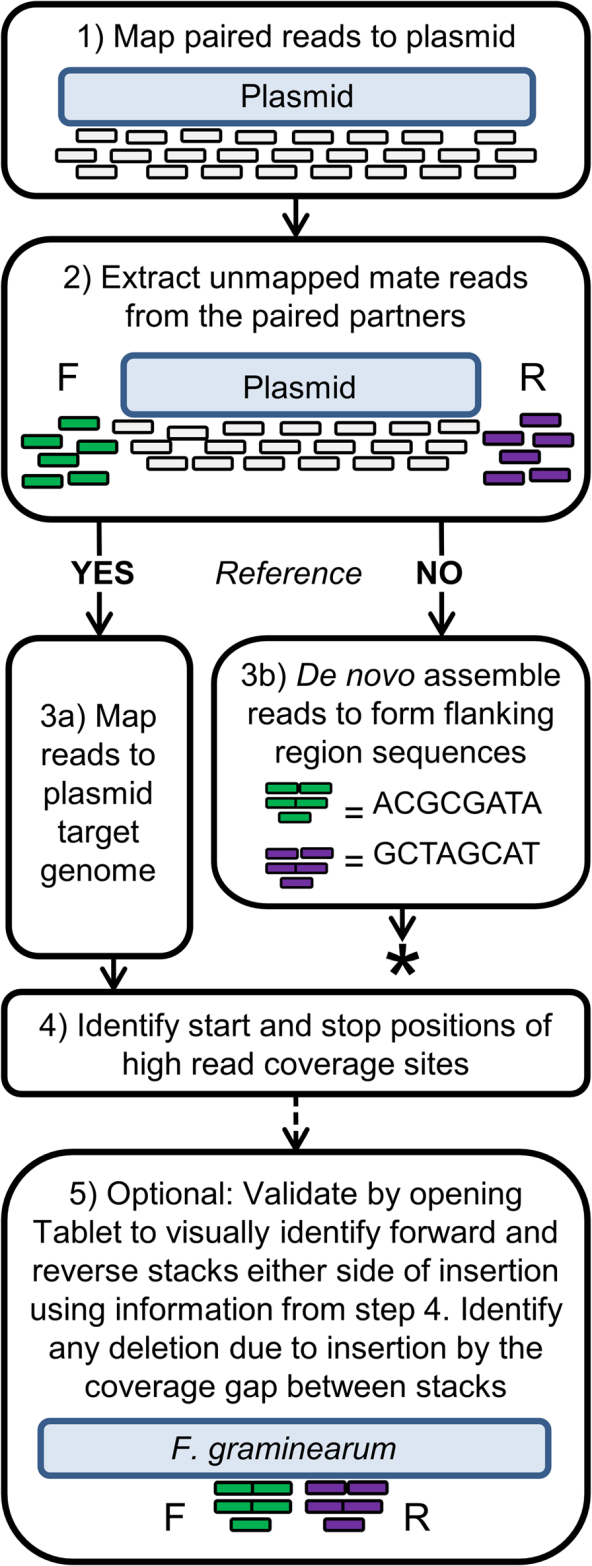
**FindInsertSeq: Sequencing workflow to identify DNA tag insertion sites and corresponding flanking genomic sequences.** Step 1, paired-end reads are mapped to the plasmid reference sequence, Step 2, extract unmapped mate reads from the paired partners that should contain sequences either side of the inserted plasmid sequence. Map the extracted mate reads to the *F. graminearum* reference genome which should form two stacks for each plasmid sequence insertion with one in the forward (F) orientation which are mated with the reads that originally mapped to the plasmid at the 5′-end and a stack with reverse (R) orientated matching reads originally mapped to the plasmid at the 3′ end. Step 3 a) the availability of reference genome is a prerequisite for this workflow. b) * if no reference genome is available *de novo* assembly of reads to identify flanking regions could be considered followed by BLAST and PCR analysis to characterise the insertion site. Step 4, to locate the stack chromosome(s) and bp positions, the mapped reads are sorted, positioned and printed to the command line. Additionally the results are printed to an individual sample text file. Open Tablet software [16] with the mapped genomic reference BAM file and proceed to the chromosome(s) and position(s) that were identified in Step 4 to visualise the insert flanking sequences and any deletion of reference sequence via the gap in coverage between the two stacks. When a reference genome assembly is either not available or both flanking sequences have not been detected due to the local incompleteness of the reference genome, then *de novo* assembly of the extracted unmapped mate reads can be used to reconstruct the sequences replacing steps 3 a through 5 with step 3 b.

**Figure 3:**
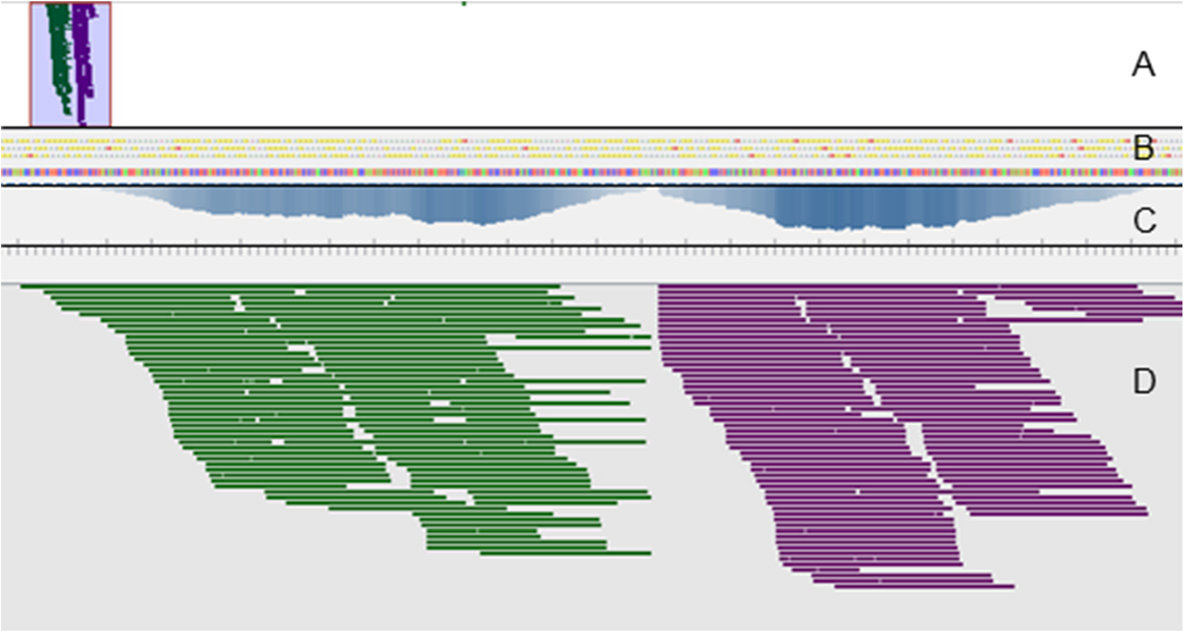
**Visualisation of a single insertion site in** ***F. graminearum*** **strain TP11.1.** Sequence reads are visualised in a screenshot using Tablet software [16]. **(A)** Sequence reads mapped to this region of the genome, **(B)** coloured lines represent the genomic sequence of the visualised region and frame translations, **(C)** sequence coverage histogram, and **(D)** mapped reads for both flanking regions and the narrow gap between the two read stacks which marks the position of the plasmid insertion. Forward green stacks and reverse purple stacks are shown.

To detect the plasmid insertion sites an analysis pipeline, called FindInsertSeq, was devised (Figure 2). The 5′- and 3′-mate sequences of the paired-end reads were mapped to the plasmid reference sequence. The pairs where both mates did not map to the plasmid were discarded. The pairs where one mate mapped to the plasmid and the other mate did not map to it, were presumed to be located at the border of the plasmid and the fusarium genomic DNA. From these desirable sequences, the latter of the pair was extracted. In these pairs the unmapped mate represented either the forward or reverse sequences that corresponded to either side of the inserted plasmid sequence (hereafter termed the flanks). These extracted unmapped mate reads were then mapped to the *F. graminearum* reference genome, already available for the strain PH-1, resulting in two stacks of reads for each plasmid sequence insertion. For the *F. graminearum* strain TP11.1 with a single plasmid insertion site in chromosome 4, sequence read stacks for the two plasmid flanks are displayed (Figure 3). The identification of loci marked by the two read stacks was achieved by sorting the mapped reads by base pair position in the sequence alignment map (SAM) file. Then a Python script using a sliding window of 300 bp to count mapped reads was used to identify where the read coverage peaked. The peak identified represents both the forward and reverse stacks and is reported by the start and stop base pair positions of the 300-bp window and the chromosome number (1–4). For mutants of interest, confirmation of the flanking regions reported by the pipeline and any deletion of reference sequence via the gap in coverage between these flanks was verified using a genome browser software such as Tablet [16]. This was used in combination with a binary alignment map (BAM) file generated by mapping the unmapped mates from the plasmid to the PH-1 reference. However, in genome regions where no reference sequence was available, or where no read stacks were found suggesting that the genomic reference was incomplete another approach was required. The extracted reads were *de novo* assembled to compile the genomic sequences flanking the inserted plasmid. This later approach was required to resolve the insertions sites of mutants DAF139 and DAF140 (see below).

### Mapping the insertions

FindInsertSeq localised the insertions in chromosome 1 for strain DAF139 and DAF140 and chromosome 4 for DAF141 and TP11.1 (Table 1). For DAF140, the tag insertion into chromosome 1 was accompanied with a small 12-bp deletion. For TP11.1, our WGS analysis confirmed the result obtained by Baldwin et al. [15] that the plasmid insertion into chromosome 4 caused a 4-bp deletion. Tag insertion in DAF139 and DAF141 were accompanied with more extensive deletions next to the insertion sites (described below). For DAF139, the insertion occurred downstream of a telomeric sequence repeat (TAACCC) [17].

**Table 1:** **Location of insertion tags and deletion events at the insertion site**

**Isolate**	**Chromosome location**	**Sequence at the 5′ end of the insertion**	Supercontig sequence deletion*	**Sequence at the 3′ end of the insertion**	**Plasmid insertion site (bp)**
DAF139	1	*TAACCC* ^*†*^ *TAACCC TAACCC TAACCC* A	1 - 503,655	GTCCAT GATACG ATGACC TTTAAC T	503,656
DAF140	1	CACATT ATAGA CTGCTA ACCTGC C	CAGGTACTGTAT	GCAGTT ACGTAG ACTGCT GAACTA A	9,177,055 - 9,177,068
DAF141	4	*Not identified*	1 - 182,475	CTTGGG GAAAGG ATCAGA GCGTGA G	182,476
TP11.1	4	TTCTTC TGGCGT TTGGCC TACAAC A	TCAT	GGTCAG CATAAG CTGGCC CCACAG C	1,519,105 -1,519,110

^*^where the deletion is large, bp positions are given.

^†^telomere sequence TAACCC given in italics.

For three of the strains the plasmid insert positions were established by mapping to the plasmid and subsequent mapping of mate reads to the reference genome. Whereas for DAF139 *de novo* assembly was necessary to identify the sequence at the 5′ end of the insertion due to an incomplete reference sequence in this part of the *F. graminearum* genome. The 5′-flanking insertion sequence was not identified for DAF141 using either approach.

### Identification of chromosome deletions and amplifications

**Figure 4:**
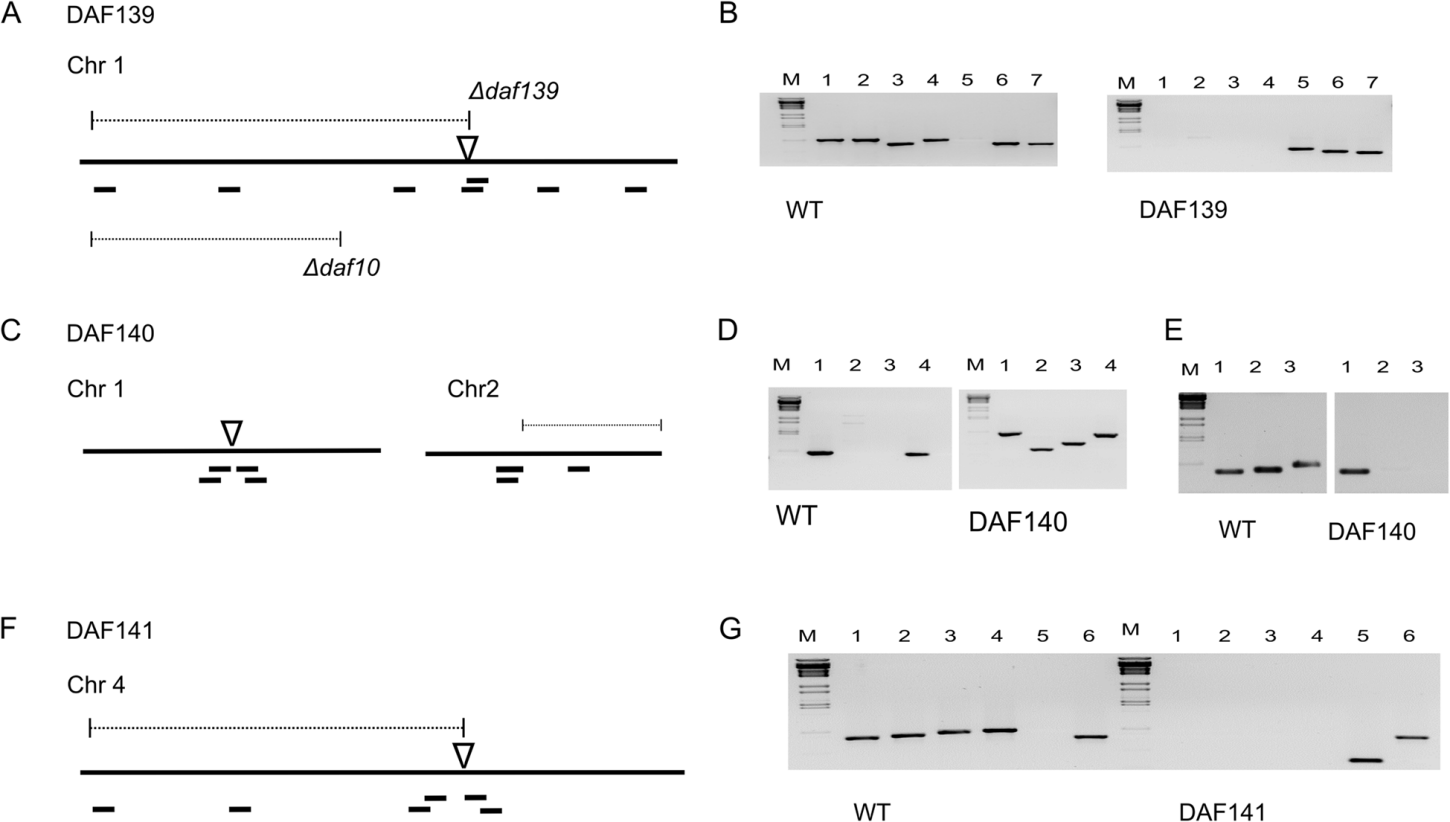
**Schematic overview of chromosomal recombination and deletion events for the three DAF mutants and verification by diagnostic PCR. (A)** DAF139 partial chromosomal deletion and insertion event. For comparison the chromosomal deletion for mutant DAF10 published earlier by Baldwin et al. [15] is indicated. **(B)** PCR analysis of chromosomal regions predicted to have either been lost or retained on chromosome 1 in strain DAF139. The wild type and DAF139 genomic DNA was amplified with oligonucleotide pairs in lanes: (1) U516/U517, (2) U518/519, (3) U550/U551, (4) U549/U521, (5) U606/U521, (6) U520/U51 and (7) U522/U523. The approximate location of the amplified PCR fragments is indicated in the schematic overview from left to right corresponding to PCR products in lane 1 to 7. **(C)** DAF140 shows a single-copy insertion in chromosome 1 and a large untagged deletion on chromosome 2. **(D)** Diagnostic PCR in lanes: (1) U533/U534, (2) U553/U602, (3) U603/U554, (4) U535/U536. Approximate location of fragments is indicated in the schematic from left (1) to right (4). **(E)** Diagnostic PCR of wild type and DAF140 chromosome 2 in lanes: (1) U540/U541, (2) U541/U502 and (3) U556/U557. Approximate location of fragments is indicated in the DAF140 schematic from left (1) to right (3). **(F)** In DAF141 three multimerised plasmids are inserted at position 182,476 bp in chromosome 4. **(G)** Diagnostic PCR of wild type and DAF141 chromosome 4 in lanes: (1) U600/U601, (2) U558/U559, (3) U545/U552, (4) U545/U546, (5) U604/U605 and (6) U547/U548. Approximate location of fragments is indicated in the schematic from left (1) to right (6). Large black lines indicate the chromosomes (not drawn to scale). Chromosomal deletions (dotted lines), plasmid insertion point (inverted open triangle). Diagnostic PCR fragments (small black lines). DNA ladder *BstE*II (M).

**Figure 5:**
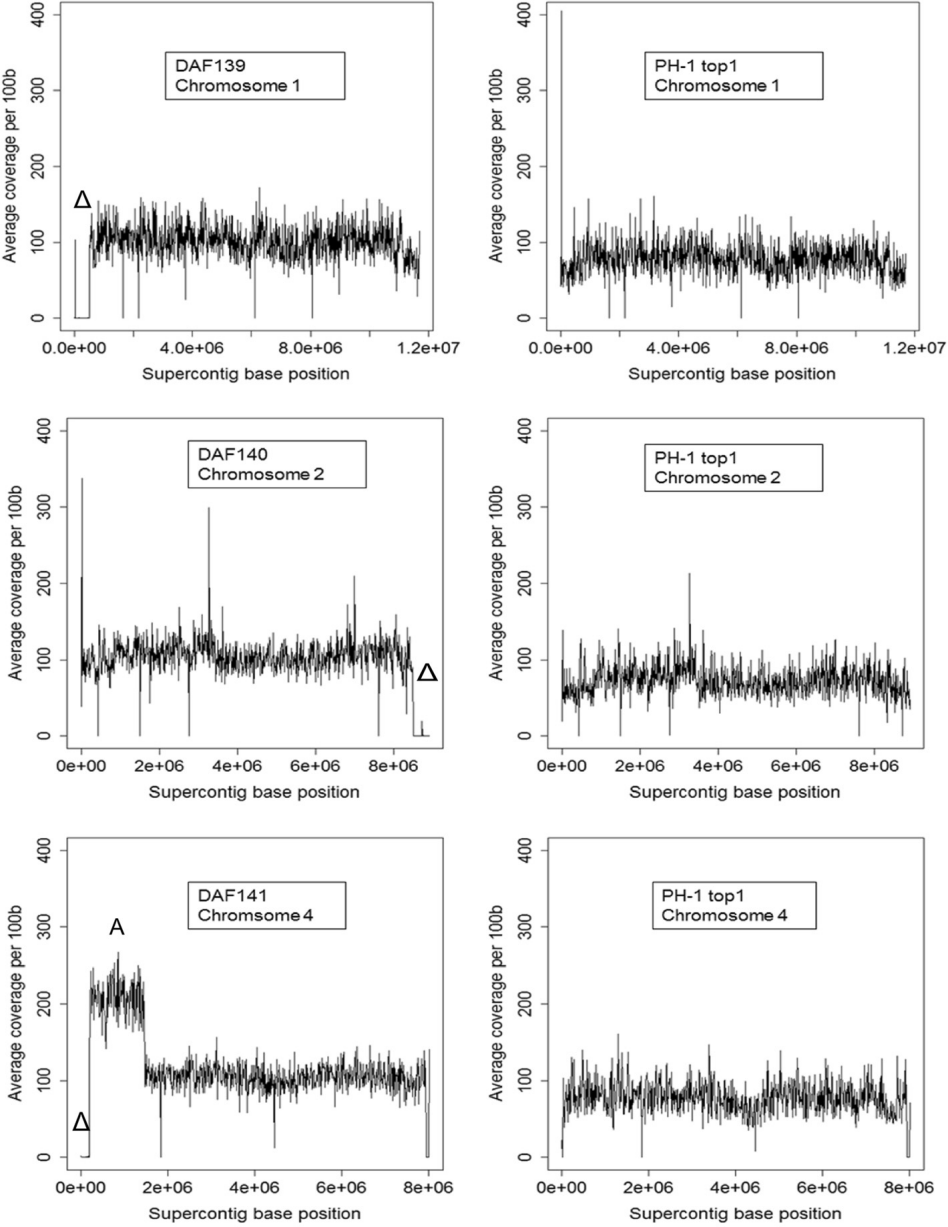
**Sequence coverage line graphs of characteristic differences at the genome level for the three DAF mutants compared to the control strain PH-1** ***top-1*** **(TP11.1).** Average numbers of sequence reads per 100 bp interval were mapped to the wild type reference genome. Top panel, DAF139 partial chromosome 1 deletion event. Middle panel, DAF140 partial chromosome two deletion event (open triangle). Bottom panel, DAF141 partial chromosome four deletion event and twofold amplification of a 1.25 MB region (A) downstream of the deletion event. The open triangle (∆) in each DAF mutant plot indicates the position of the loss in genomic sequence. For each mutant, the corresponding chromosome in the PH-1 *top1* mutant (TP11.1) is depicted as a control.

The sequencing of the mutant genomes permits further qualitative analysis which may be missed using molecular biology methodologies resulting in falsely linking a retrieved gene sequence to a phenotype. For each of the DAF mutants, the obtained genomic reads were mapped to the *F. graminearum* reference genome (Figure 4). This analysis detected additional deletion and/or copy number variations of genomic sequence associated with the plasmid insertion process. Other formal possibilities by which variation could have been introduced are cellular stress responses induced by the transformation method, growth and storage conditions. In the TP11.1 mutant no detectable amplifications or deletions other than at the plasmid insertion site were detected. For DAF140, the DNA gel blot analysis had predicted a single plasmid insertion event into the genomic DNA (Figure 1). However, this further sequence analysis revealed that besides the insertion in chromosome 1, an additional untagged chromosomal deletion had occurred. This untagged deletion event had resulted in the loss of 415,100 bp at the 3′-end of chromosome 2 predicted to contain 177 genes (Additional file 1: Table S2). For DAF141, the DNA gel blot analysis suggested the presence of potentially two copies of the plasmid (Figure 1). The WGS analysis revealed that the single insertion site was accompanied with a 182.4-kb deletion close to the 5′-end of chromosome 4 resulting in the loss of 72 genes upstream. However, further inspection of the DAF141 WGS data also identified a twofold amplification of 1.25 Mb immediately downstream of the deletion event (Figure 5). For DAF139, the DNA gel blot analysis had suggested a highly complex insertion event. The plasmid insert in DAF139 resulted in the loss of 503.6 kb at the 5′-end of chromosome 1 but had left the telomere intact. The deleted genomic region in DAF139 is predicted to contain 207 genes (Table 2 and Additional file 2).

**Table 2:** **Deletion of gene regions in the four mutants and numbers of genes lost**

**Strain**	**Chromosome**	**Position of sequence loss (bp)**	**Number of genes contained within the lost regions**
DAF139	1	1 - 503,655	207
DAF140*	2	8,496,503 - 8,911,601	177
DAF141	4	1 - 182,475	72
TP11.1	None	None	None

*For DAF140, the plasmid vector insertion occurred in chromosome 1 (see Table 1). Chromosome 2 was affected by an untagged gene deletion event.

### Estimation of plasmid copy number

**Figure 6:**
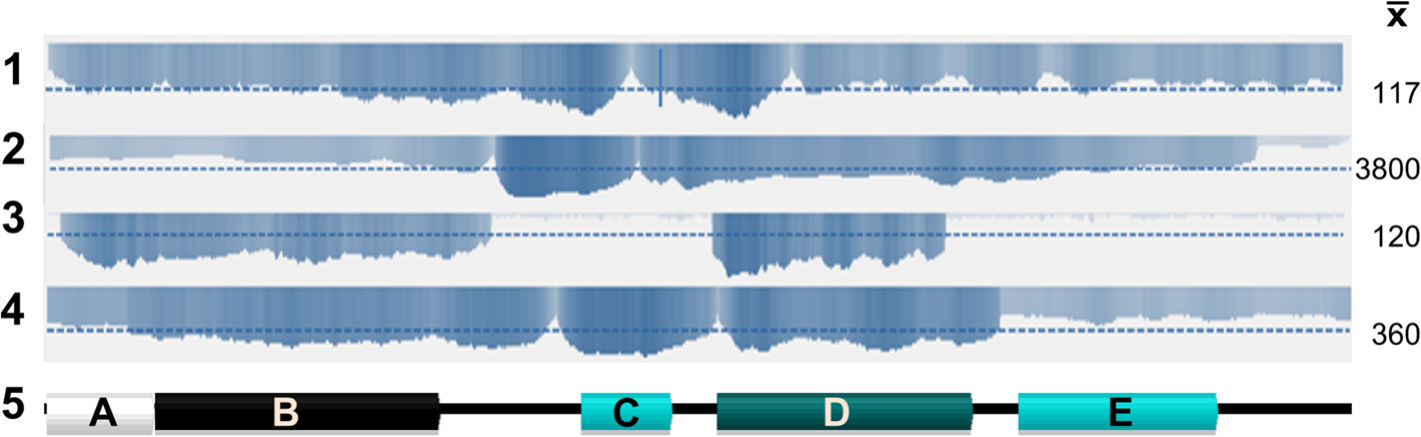
**Coverage histogram of reads mapped to the inserted plasmid in the four mutants.** Rows are: (1) TP11.1 (2) DAF139 (3) DAF140 and (4) DAF141. The experimentally obtained plasmid average read coverage $\left(\overline{\mathrm{X}}\right)$ for each mutant is given on the far right. The reference genome read coverage for TP11.1 is 86.6. Row 5 depicts the genetic elements of the insertion plasmid. **(A)** *trpC* promoter, **(B)** hygromycin-phosphotransferase, **(C)** F1 origin of replication, **(D)** ampicillin and **(E)** *E. coli oriC*.

For two of the DAF mutants, DAF 141 and DAF139, the plasmid copy number inferred from the DNA gel blot analysis and the number of insertion sites detected within the *F. graminearum* genome by WGS analysis did not precisely match. Therefore an estimation of plasmid copy number by WGS was attempted for all four mutants. To achieve this, the coverage histograms of TP11.1 and the three DAF mutants were plotted across the whole plasmid sequence of the vector pHYG1.4 (Figure 6) to identify whether there had been recombination of the plasmid resulting in incomplete plasmid sequence insertion. Average calculated genome wide coverage for the TP11.1 strain was 86.6 vs. the experimental observed value of 117 for the mapped reads to the plasmid sequence. This suggested an approximate 1.35× plasmid length insertion in TP11.1, however only 1× plasmid length insertion was detected from the *de novo* assembly. This result suggests that this WGS–plasmid alignment approach for the estimation of plasmid copy number is fairly accurate.

Coverage of the three DAF strains across the plasmid sequence was found to be variable with either some segments absent and/or other segments with increased read density indicating multiple copies of plasmid sequence at the insertion site. In DAF139 the plasmid had an average observed coverage of 3800 suggesting an insertion sequence length of approximately 36 plasmids based upon a calculated read-coverage of 104. However some segments of the sequence were over represented. A similar case was found for sample DAF141 with an average coverage of 360 but a calculated coverage of 114 suggesting a length of three plasmids inserted into the genome. PCR amplification of the concatenated plasmid is hindered by the large size of the concatemer and due to the fact that it consists of repeated elements. Similarly, *de novo* assembly is hampered due to the repetitive nature of the concatemer that had formed.

The data obtained for DAF140 suggests a single plasmid insertion of 50% of the length of the sequence with an observed coverage of 120 in the regions where coverage was evident vs. a calculated coverage of 119. Both *TP11.1* and DAF140 insertion sequences were validated using *de novo* assembly and support the reported findings. Interestingly, the plasmid gene cassettes noticeably amplified in DAF 139, DAF140 and DAF141 include the resistance marker hygromycin-phosphotransferase and the bacterial ampicillin resistance gene while the *E. coli oriC* replication sequences of the plasmid vector were found to be less frequently amplified. Collectively, these data indicate that multiple plasmid concatenations at a single insertion site had occurred in both the DAF139 and DAF141 mutants.

### Experimental verification of insertions and chromosomal deletions by PCR

To demonstrate that the computationally predicted plasmid insertion sites can be verified in the laboratory, insertions and chromosomal deletions were confirmed using a PCR based approach. This test also excluded the possibility of translocations of affected chromosomal regions into other chromosomes. Primers were designed to detect the presence or absence of ~0.7-kb diagnostic fragments across the modified chromosomes. Plasmid insertion sites were verified by designing oligomer pairs where one primer site lies within the inserted plasmid and the corresponding primer site lies within the adjacent chromosomal neighbourhood. For DAF139, four diagnostic fragments located in the region of chromosome 1 predicted to be deleted, could not be amplified while amplification of wild type (WT) DNA produced the expected fragments (Figure 4). Two further diagnostic fragments upstream of deleted chromosomal region were amplified from both DAF139 and WT DNA. A diagnostic fragment originating from a primer site located in the inserted plasmid and from the adjacent chromosomal neighbourhood was only amplified in DAF139 but not in WT DNA. The results verified the computationally predicted chromosome modifications. For DAF140 a similar approach confirmed the plasmid insertion site in chromosome 1. In this mutant whole-genome analysis had also revealed an untagged chromosomal deletion in chromosome 2. The deletion and its extent were confirmed using three diagnostic fragments. For DAF141 plasmid insertion and a chromosomal deletion was confirmed for chromosome 4. The exact plasmid insertion sites were further confirmed by sequencing the diagnostic fragments obtained using the plasmid specific oligomers. These data independently confirm the insertion and chromosomal deletion results predicted using our whole-genome sequencing and bioinformatics data analysis pipeline described in this study.

### Virulence genes identified close to the insertion points or in chromosomal deletions

The adjacent gene(s) close to the plasmid insertion points and genes contained in the chromosomal regions deleted in mutants DAF139, DAF140 and DAF141 were analysed by BLAST analysis against the Pathogen-Host Interactions (PHI) database (http://www.phi-base.org) composed of experimentally verified, virulence and effector proteins from bacteria, fungi and oomycete pathogens that infect a wide range of hosts [18]. PHI-base associates gene and protein information with published fungal mutant phenotypes. These phenotypes include altered virulence phenotypes (increased and reduced virulence, loss of pathogenicity), unaffected pathogenicity, and lethal (essential gene). In the deleted regions of DAF139, DAF140, DAF141 the BLAST analysis revealed the presence of 44, 27 and 13 PHI-base homologs with the phenotype “altered virulence” at E ≤ 0.0001 (Additional file 2). We conclude that the absence of these functional homologs of virulence-associated genes is responsible for the pathogenicity defect of the three mutants on wheat. The BLAST analysis also revealed that in the three mutants a total of five putative transcription factor genes had been deleted that were previously implicated to be essential genes in *F. graminearum*. In a genome-wide single gene deletion study of transcription factors no successful reverse genetics mutants could be obtained [19]. These genes are in DAF139, FGSG_00144 (GzZC306), in DAF140, FGSG_16054 (GzZC062), FGSG_16056 (GzZC089) and in DAF141, FGSG_06448 (GzZC029), FGSG_17682 (GzZC129). The absence of these transcription factor genes was confirmed by PCR amplification using the wild-type strain PH-1 as the positive control (Additional file 1: Figure S3). We conclude that the five transcription factor genes are not required for growth in the characterised *F. graminearum* insertion mutants generated in the strain PH-1.

### Random sampling

**Figure 7:**
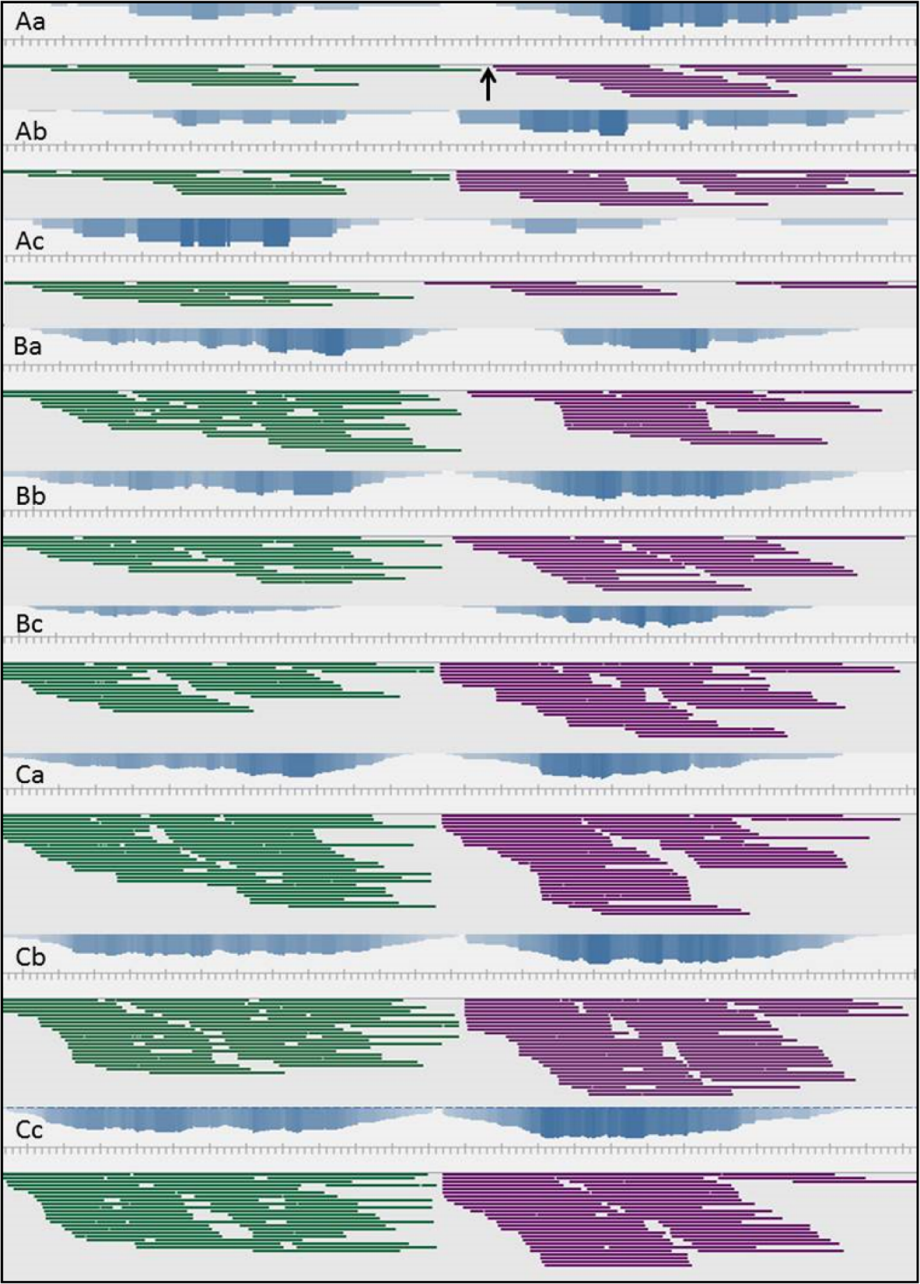
**Random data sampling to determine the minimal genome coverage required for insertion point characterisation in** ***F. graminearum*** **strain TP11** ***.*** **1.** (Aa-Ac) Sampling 1 with three reduced data replicates of 10% each. The deletion sequence between 5′ and 3′ read stacks is represented with an arrow in Aa. (Ba-Bc) Sampling 2 replicates with 25% of data. (Ca-Cc) Sampling 3 replicates with 50% of data. The average coverage for each data set in A, B and C at the insertion region is 10, 24, and 48 respectively. The original data set had an approximate coverage of 86.

Subsampling of the Illumina sequence read dataset was computationally performed for the TP11.1 mutant strain using three different seed values. The original data set had an approximate coverage of 86. The seqtk program was used to produce three technical replicates for 10% (3,205,728 reads), 25% (8,014,318), and 50% (16,028,636 reads) of the total reads. The average genome coverage for each data set is 10, 24, and 48, respectively. Visual inspection of the datasets (Figure 7) identifies that the insertion tag can unambiguously be identified in all three replicates for the 25% and 50% subsamples but not for the 10% subsample. We conclude that a 22× coverage obtained by the Illumina HighSeq 2000 sequencing platform is sufficient for *F. graminearum* to identify insertion tags.

## Discussion

In this study we setup and describe a bioinformatics workflow called FindInsertSeq (Figure 2) to efficiently characterise forward genetics mutants created by plasmid insertion in the filamentous plant pathogen *Fusarium graminearum* using Illumina whole-genome sequencing approach with paired-end reads. The four mutants selected showed a reduced virulence phenotype on their host wheat. The mutants were selected to include low and complex insertion events visualised by DNA blot analysis. Using the FindInsertSeq pipeline we were able to rapidly identify DNA tag insertion points to the nucleotide level for the control strain TP11.1 and three novel disease attenuated Fusarium (DAF) mutants generated for this study.

The principle of the WGS-FindInsertSeq analysis approach is to map sequence reads to an insertion vector and use the mate that aligns to the genomic reads to identify the insertion point in the genome. FindInsertSeq can also be used to discriminate between single insertion and complex tandem insertion events in a mutant where a reference sequence is available for the species or could be adapted where a reference genome is lacking (see Figure 2, step 3 b). Thirdly, when *de novo* mapped read coverage comparisons of mutant and wildtype DNA were included in the method, this revealed the existence of further unexpected recombination events, such as chromosomal deletions and amplifications. To our knowledge this study, for the first time, demonstrates the analyses of complex insertion events on the whole-genome level in a pathogenic filamentous fungus of medium 37-Mb-genome size. The predictions made using the FindInsertSeq pipeline were independently confirmed through diagnostic PCR analysis.

Current laboratory-based methods to determine insertion points such as TAIL-PCR [20] and plasmid rescue [1] can, in most cases, only successfully be used to identify the insertion event where a single copy of the insertion tag has occurred. Many research groups over the past 20 years have created mutant libraries of which only a small number of single-copy insertion mutants were successfully analysed [21,22]. Such insertion mutant collections are now amenable for further analysis using WGS analysis and the FindInsertSeq pipeline.

For sequencing we used the Illumina HiSeq2000 platform, which is currently the standard high-throughput sequencing method [6,23]. Sequencing of an 800-bp fragment library using 100-bp paired-end reads generated ~100× fold *F. graminearum* genome coverage when four samples were pooled across one sequencing lane. The size of the fragment library is an important factor. If the fragment size exceeds the insertion vector length, an offset of the insertion point will occur. This offset can be calculated as mean library fragment length minus insertion vector length.

The choice of mapping software can influence the success of the workflow. A comparison of Novoalign [24], Bowtie2 [25] and BWA [26] mappers using the default settings showed that only the first two successfully mapped the insert (RK, data not shown). However, the BWA aligner did not map the reads to the correct position in three of the four strains analysed. This result was attributed to the loss of direction of the paired reads because both stacks of the unmapped mate reads were represented in the forward orientation.

An alternative strategy was considered via *de novo* genome assembly followed by applying BLAST to identify the insertion site of the plasmid. However such an assembly-based approach was not implemented due to the higher computational resources required to assemble each mutant compared to mapping first to a short plasmid sequence, followed by aligning a small subset of reads to the reference genome. For species with larger genomes i.e. plants, plant-infecting insects or pathogenic protists *de novo* assembly is challenging and often dependent on many non-intuitive parameters such as k-mer value selection. In addition the output from the mapping approach (see Figure 3) provides three visually compelling features that the insertion site has been correctly identified. The characteristic features are: two flanks with a similar number of aligned reads separated by a clear gap where the plasmid inserted and deleted a variable number of nucleotides.

The FindInsertSeq workflow currently advises visual inspection using the Tablet software [16] as an optional step for mutants of further interest, to inspect forward and reverse sequence stacks either side of the insertion point (Figure 2 step 5) and identify the deletion and flank sequence to 1 bp. Currently only an approximate location of the insertion is given for screening purposes of the first and last base of the highest read coverage peak using a set window size (default 300 bp). This location is written to output (step 4) but surmises the region of the genome in which the insertion took place. This approach permits the biologist to experimentally confirm the insertion point using a PCR-based analysis.

Randomly down-sizing the sampled genomic read coverage for the control mutant strain TP11.1 revealed that only 22 x genomic coverage is required to identify the insertion site with a cost incurred per sample of approximately $200 including all associated cost such as DNA preparation and library construction. Sequencing methods are evolving rapidly and as a consequence associated costs are expected to drop even further.

Many fungal genomes are of similar size to *F. graminearum* and have a similar GC content. A WGS-FindInsertSeq approach can identify multiple locations of plasmid insertions. However, this may prove more difficult to apply in fungal eukaryotes such as *Magnaporthe oryzae* for which 10 % repetitive DNA is reported [27]. If the insertion event flanking sequence contains repeat sequences, mapping will be ambiguous.

Transformation events may result in reshuffling and partial amplification of the insertion tag [22]. We found this strikingly to be the case for the mutant strain DAF140. The coverage histogram (Figure 6) revealed that the resistance marker gene encoding hygromycin-phosphotransferase (*hph*) was highly amplified during vector plasmid integration as well as the ampicillin gene cassette. These events could be favoured by DNA structural elements in the vector influencing the outcome of recombination events. The amplification of the *hph* gene is likely to be selected for when growing the transformants on hygromycin-containing medium.

In addition to the pipeline described in Figure 2 we investigated situations where only a single flanking sequence was identified. This outcome suggested further genomic alterations might have occurred. In these cases all genomic reads from the mutant were mapped back to the reference. This approach successfully revealed additional tagged and untagged chromosomal deletions and amplifications events in the mutants (Figure 5). The extent of the chromosomal deletions is surprising and suggests that larger parts of the *F. graminearum* genome are not required for growth *in vitro* and / or during the protoplast transformation and regeneration procedure used in this study. Similar results were found for two *F. graminearum* mutants, called Z43R606 and DAF10, where the occurrence of large chromosomal deletion events was reported earlier [21,28]. In both studies a combination of plasmid-rescue and Sanger single-read sequencing was used to characterise the mutants. In Z43R606 a genomic region of 220 kb containing at least 92 genes was deleted, whilst in DAF10 a genomic region of 350 kb containing 150 genes was deleted.

The molecular analysis of Z43R606 revealed a deletion of a genomic region at the vector insertion site, including the gene clusters required for the biosynthesis of aurofusarin and zearalenone. The authors deliberately recreated the deletion event using a reverse genetics approach to demonstrate that a large region of genomic DNA can be efficiently deleted in *F. graminearum* by double homologous recombination [28]. In the DAF10 study, the unintentional deletion of a large region of genomic DNA permitted the authors to report that the loss of the *TRI1* gene involved in mycotoxin biosynthesis was responsible for the virulence defect on its host. Interestingly, the mutant DAF139 recreated the DAF10 mutant and extended the deleted region by an additional 57 genes (153 kb). Taken together, these findings show that large chromosomal regions can systematically be deleted in *F. graminearum* to understand genes sets and genome organisation. In *S. cerevisiae,* such an approach is currently being used to build a minimalist yeast genome within the Sc2.0 project [29].

The deleted chromosomal regions in the analysed mutants identified a total of 456 genes that when deleted from the *F. graminearum* genome are not required for life and are thus unlikely to make good fungicide targets. Within this gene set were five putative transcription factors that were earlier proposed to be essential for life in *F. graminearum* [19]. Son and colleagues [19] tested in a genome-wide gene deletion study 657 putative transcription factors, but no gene deletion mutants could be obtained for these five genes. While it is possible that the absence of these proteins is compensated by the absence of additional proteins within the deleted chromosomal regions, further experiments are needed to elucidate their function.

## Conclusions

This study demonstrates that whole-genome sequence analysis is an efficient tool to characterise forward genetics mutants in the filamentous pathogenic species *F. graminearum*. Surprisingly in some cases the DNA tag insertion events are accompanied with large untagged chromosomal deletions while in other cases a straight-forward insertion event could be confirmed. This fact suggests that the FindInsertSeq method identifies DNA tag insertion points and other genomic recombination events such as chromosomal deletions and DNA amplifications. Large scale chromosomal deletions can quickly identify the non-essential gene set or host-specificity genes not essential for pathogenic life. The minimal sequencing coverage required in *F. graminearum* to survey accurately the genome to a single base level for recombination events was determined to be 22× fold. FindInsertSeq will also allow full characterisation of recombinant filamentous fungal isolates overexpressing heterologous proteins for research and/or industrial scale commercial applications.

## Methods

### Fungal growth, mutagenesis, DNA preparation, sequencing and PCR analyses

Fungal strains were routinely cultured as described [30]. *F. graminearum* strain PH-1 was used to generate insertional mutants using the plasmid pHYG1.4 (Additional file 1: Figure S1) and tested for virulence on the susceptible spring wheat cultivar Bobwhite using established methods [15]. Genomic fungal DNA for sequencing was extracted using the CTAB protocol [31] and purified using a Qiagen Kit (Qiagen Ltd, Crawley, West Sussex, UK). High-quality genomic DNA was then submitted to the TGAC (Norwich, UK) for generation of a 0.8-kb fragment library. The Illumina HiSeq 2000 sequencing platform (San Diego, CA) was used to produce 100-bp paired-end reads [6]. Primer design, PCR conditions and single-read sequencing of PCR products followed the protocols described by Baldwin et al. [21]. Primer sequences used for gene detection and experimental verification of chromosomal deletions are listed in Additional file 1: Table S3 and S4. The DNA ladder *BstE*II used for the diagnostic PCR was obtained from New England Biolabs, Hitchin, UK.

### Mapping and assembly

Raw reads were not pre-processed. Seqtk [32] was used to subsample randomly reads to three technical replicates of 10%, 25% and 50%.

For the plasmid mapping approach, the software Novoalign3 [24], Bowtie2 [25] and BWA [26] were used to align the sequencing reads to the plasmid sequence (pHYG1.4) using default parameters, followed by the removal of unmapped reads that were the paired partners of mapped reads using SAMtools [33]. The extracted forward read set and reverse read set correspond to sequences either side of the inserted plasmid. Each set was then mapped to the *F. graminearum* reference sequence (MIPS version FG3.2, ftp://ftpmips.gsf.de/fungi/FGDB/v32/) [34]. Alignments were converted from the sequence alignment map (SAM) to binary alignment map (BAM) format. BAM files were then sorted and indexed using SAMtools [33]. Visualisations were done using the Tablet software [16] and the Integrative Genomics Viewer (IGV) [35]. Coverage of mapped raw reads to the *F. graminearum* reference were calculated using DELLY [36] and R [37] was used to plot the resulting coverage comparisons. Detailed instructions on running the FindInsertSeq workflow are available at https://github.com/Rothamsted/AppliedBioinformatics/tree/master/FindInsertSeq.

Each DAF mutant genome was assembled with SOAPdenovo2 [38]. For mutants with single insertions a k-mer value of 61 was used to identify the integrity of the plasmid insertion event and the flanking sequences. For mutants with multiple insertions, a range of k-mer values were tested, i.e. k-mer 61, 63, 65, 67, 69, 71, 81, 91. By this approach only small flanking sequences were assembled to the plasmid sequence. These small flanking sequences were sufficient to verify the insertion position obtained by the standard pipeline. The resulting assemblies were used to confirm the orientation, bp deletion, and plasmid insertion copy number where applicable (not successful for multiple copy plasmid insertions) by alignment to the reference sequence *Fusarium graminearum* PH-1 genome and the plasmid sequence within Geneious version 7.1.6 created by Biomatters (available from http://www.geneious.com/) using LASTZ [39].

### Analysis of the gene types lost through genomic deletion

The *F. graminearum* reference sequence (MIPS version FG3.2, ftp://ftpmips.gsf.de/fungi/FGDB/v32/) was downloaded. Gene IDs within the deleted chromosomal regions of the insertion mutants were extracted using Geneious version 7.1.5 created by Biomatters available from http://www.geneious.com. The PHI-base database version 3.6 (May 2014 release) was downloaded from http://www.phi-base.org/ as a FASTA file [18]. A custom BLAST database was setup and implemented on a TimeLogic® Tera-BLAST™ DeCypher system (Active Motif Inc., Carlsbad, CA). For each gene the top BLAST hit (E-value of ≤10E-4) with annotation including the experimentally tested phenotypes was extracted (Additional file 1: Table S5 and Additional file 2).

### Data access

The reference genome information for *F. graminearum* strain PH-1 (NRRL 31084) is available at GenBank under accession AACM00000000 [12]. Whole-genome sequencing information for the strains described in this study is available at the Sequence Read Archive (SRA). The accession numbers are: ERS430784 (DAF139), ERS430785 (DAF140), ERS430786 (DAF141) and ERS430787 (TP11.1).

## Additional files

Additional file 1: Figure S1. Map of plasmid pHYG1.4 and oligos used for *hph* probe generation. **Figure S2.** Deletions overview in mutants reported in the literature and in this study. **Figure S3.** Absence and presence of five putative transcription factors in fusarium insertion mutants and WT strain PH-1. **Table S1.** Obtained sequence coverage of mutants. **Table S2.** Deleted genes in the three DAF mutants not essential for life. **Table S3.** PCR fragment sizes. **Table S4.** Primer sequences. **Table S5.** Five transcription factors which do not have an essential gene function. Additional file 2:
**List of genes deleted in mutants DAF139, DAF140, DAF141, DAF10, Z43R606, and respective PHI-base BLAST results.**

## Abbreviations

BAM: Binary alignment map
DAF mutant: Disease-attenuated Fusarium mutant
*Hph*: Hygromycin-phosphotransferase
PCR: Polymerase chain reaction
SAM: Sequence alignment map
WGA: Whole-genome analysis
WGS: Whole genome sequencing
WT: Wildtype
