# Supplementary material for: Whole-genome analysis of Fusarium graminearum insertional mutants identifies virulence associated genes and unmasks untagged chromosomal deletions
Source: BMC Genomics. 2015 Apr 3;16(1):261. doi: 10.1186/s12864-015-1412-9 (PMC4404607; doi:10.1186/s12864-015-1412-9)
Supplement: Additional file 1: Figure S1. — Map of plasmid pHYG1.4 and oligos used for hph probe generation. Figure S2. Deletions overview in mutants reported in the literature and in this study. Figure S3. Absence and presence of five putative transcription factors in fusarium insertion mutants and WT strain PH-1. Table S1. Obtained sequence coverage of mutants. Table S2. Deleted genes in the three DAF mutants not essential for life. Table S3. PCR fragment sizes. Table S4. Primer sequences. Table S5. Five transcription factors which do not have an essential gene function. [file 12864_2015_1412_MOESM1_ESM.docx]

# Additional file 1

Figure S1


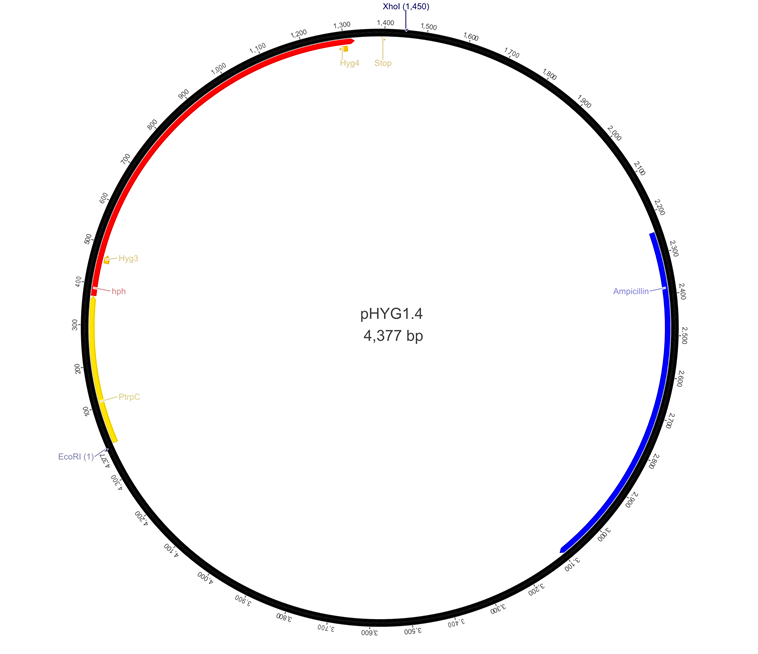


**Figure S1:** Map of plasmid pHYG1.4 and oligos used for hph probe generation

The vector used for insertional mutagenesis pHYG1.4 encodes the *E. coli* hygromycin phosphotransferase resistance protein (Uniprot P00557) using the *A. nidulans trpC* promoter for expression *in F. graminearum*. Hyg3 and Hyg4 primers were used to produce an 855-bp-long hph probe for Southern analysis. Single *EcoR*I and *Xho*I restriction sites used are indicated.

Figure S2


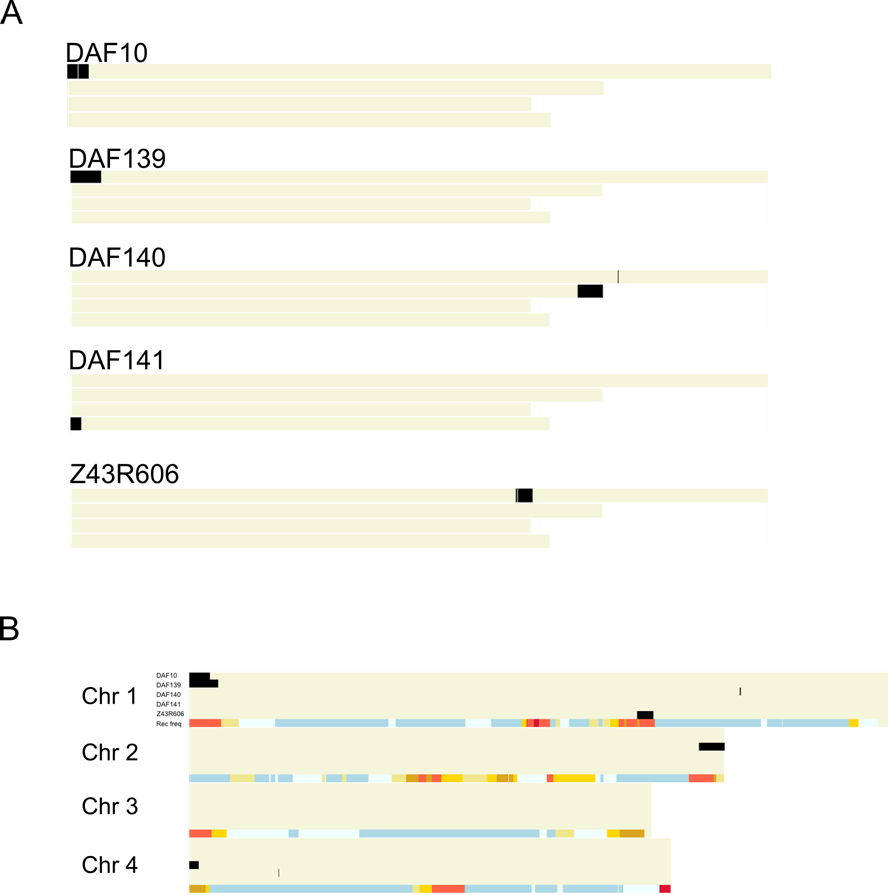


**Figure S2**: Deletions overview in mutants reported in the literature and described in this study

(A) Five insertion mutants are depicted using OmniMapFree software [1]. DAF139, DAF140 and DAF141 were analysed in this study. The two mutants DAF10 and Z43R606 were described earlier [2, 3]. For each mutant all four chromosomes are depicted. Deleted genes are indicated by black bars and large chromosomal deletions are indicated by black boxes. (B) Chromosome-by-chromosome comparison for the five mutants. Track 6 for each chromosome depicts the recombination frequency (cM/27 kb) across the chromosome using a colour gradient from white (lowest) to crimson (highest) [1, 4].

Figure S3


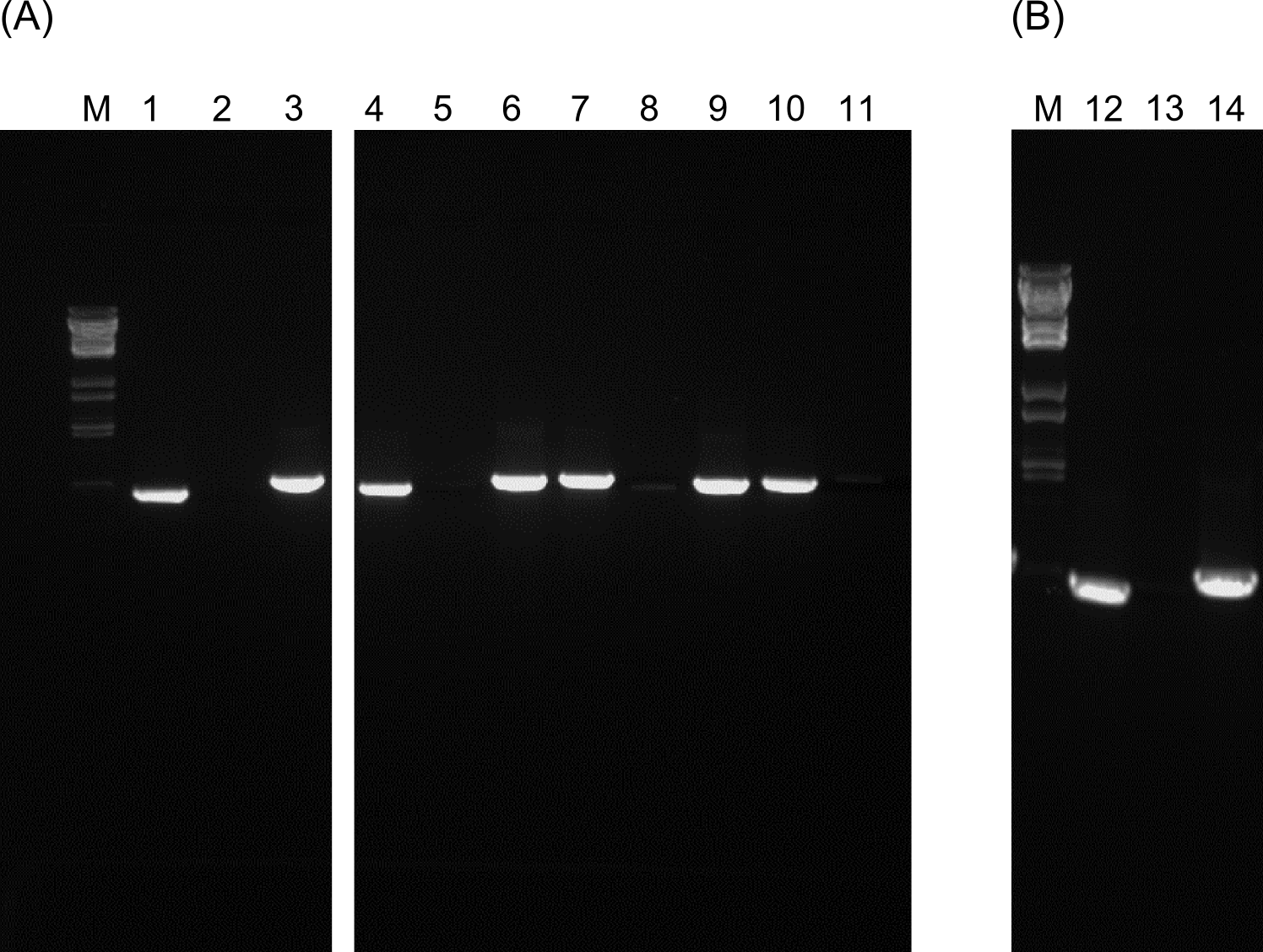


**Figure S3:** Absence and presence of five putative transcription factors in fusarium insertion mutants and WT strain PH-1

(A) Annealing temperature of 56 C during PCR (B) Annealing temperature of 60 C during PCR. M indicates the DNA ladder *BstE*II. PCR reactions are given in the table beneath. All reactions were analysed on a 1% Agarose gel in the presence of Ethidium bromide stain (0.5 µg/ml).

Oligomers U611/U612 amplify an internal *TOP1* gene amplicon of 801 bp and where used to test the integrity of the fusarium mutant genomic DNAs.

Loadings for Figure S3

| Lane | DNA Template | Oligo 1 | Oligo 2 | Amplicon |
| --- | --- | --- | --- | --- |
| 1 | PH-1 (WT) | U613 | U614 | FGSG_00144 |
| 2 | DAF139 | U613 | U614 | FGSG_00144 |
| 3 | DAF139 | U611 | U612 | *TOP1* control gene |
| 4 | PH1 | U617 | U618 | FGSG_16056 |
| 5 | DAF140 | U617 | U618 | FGSG_16056 |
| 6 | DAF140 | U611 | U612 | *TOP1* control gene |
| 7 | PH1 | U619 | U620 | FGSG_06448 |
| 8 | DAF141 | U619 | U620 | FGSG_06448 |
| 9 | DAF141 | U611 | U612 | *TOP1* control gene |
| 10 | PH1 | U621 | U622 | FGSG_17682 |
| 11 | DAF141 | U621 | U622 | FGSG_17682 |
| 12 | PH1 | U615 | U616 | FGSG_16054 |
| 13 | DAF140 | U615 | U616 | FGSG_16054 |
| 14 | DAF140 | U611 | U612 | *TOP1* control gene |

Table S1

**Table S1:** Obtained sequence coverage of mutants

| **Sample name** | **Mean library fragment size** | **Clusters** | **Total Reads** | **Total Data (Gbp)** | **Q30 data (Gbp)** | **Average Q30 read 1 (bp)** | **Average Q30 read 2 (bp)** | **Estimated Genome Size (Mbp)** | **Average Coverage (all data)** | **Average coverage (Q30 data)** |
| --- | --- | --- | --- | --- | --- | --- | --- | --- | --- | --- |
| DAF139 | 427 | 19,358,460 | 38,716,920 | 3.87 | 3.87 | 101 | 99 | 37 | 104.6 | 104.6 |
| DAF140 | 437 | 22,052,504 | 44,105,008 | 4.41 | 4.41 | 101 | 99 | 37 | 119.2 | 119.2 |
| DAF141 | 449 | 21,087,056 | 42,174,112 | 4.22 | 4.22 | 101 | 99 | 37 | 114.0 | 114.0 |
| TP11.1 | 408 | 16,028,635 | 32,057,270 | 3.21 | 3.21 | 101 | 99 | 37 | 86.6 | 86.6 |

Table S2

**Table S2.** Deleted genes in the three DAF mutants not essential for life.

| **Mutant** | **Genes lost** | **PHI-base** homologs** | **Kb deleted** | **Chromo-some** | **PHI-base entry with altered virulence** | **Suggested to cause lethality** |
| --- | --- | --- | --- | --- | --- | --- |
| DAF10^*^ | 150 | 57 | 350 | 1 | 36 | 0 |
| DAF139 | 207 | 81 | 503 | 1 | 44 | 1 |
| DAF140 | 177 | 51 | 415 | 2 | 27 | 2 |
| DAF141 | 72 | 28 | 182 | 4 | 13 | 2 |
| Z43R606^**^ | 92* | 57 | 220 | 1 | 20 | 0 |

^*^ DAF10 is described by [2].

^**^ Z43R606 is decribed by [3]. Authors describe that the deleted region contains 52
 putative genes, but a higher number was obtained from the latest *F. graminearum* genome reference (MIPS version FG3.2, ftp://ftpmips.gsf.de/fungi/FGDB/v32/)

^***^ Analysed using the entries in the pathogen-host interactions (PHI) database

Table S3

**Table S3.** PCR fragment sizes.

| **Primer pair** | **Strain** | **PCR amplicon expected** |
| --- | --- | --- |
| U545 / U552 | DAF141 | - |
| U545 / U552 | WT | 670 bp |
| U549 / U521 | DAF141 | - |
| U549 / U521 | WT | 700 bp |
| U550 / U551 | DAF140 | - |
| U550 / U551 | WT | 578 bp |
| U553 / U602 | DAF140 | 300 bp* |
| U553 / U602 | WT | - |
| U556 / U557 | DAF140 | - |
| U556 / U557 | WT | 697 bp |
| U558 / U559 | DAF141 | - |
| U558 / U559 | WT | 644 bp |
| U600 / U601 | DAF141 | - |
| U600 / U601 | WT | 614 bp |
| U603 / U554 | WT | - |
| U603 / U554 | DAF140 | 417 bp |
| U604 / U605 | DAF141 | 254 bp* |
| U604 / U605 | WT | - |
| U606 / U521 | DAF139 | 600 bp* |
| U606 / U521 | WT | - |

* PCR amplicon can only be expected if one oligomer binds to the insertion vector sequence and the other one to a site in the genomic fusarium DNA

Table S4

**Table S4:** Primer sequences

| **Primer Name** | **Sequence** |
| --- | --- |
| U516 | CATGTCCGCACGTTCCATAA |
| U517 | CCCCCGATAGAAGATCAAGT |
| U518 | AATGACGCGCTTGGCAAGAA |
| U519 | ATTGGACTGAAAAGGCCGAG |
| U520 | CCACTGACACTCTGAATCC |
| U520 | GTCCATGATACGATGACCTT |
| U521 | GTTGCAGCTGTTACAGTTGC |
| U522 | TGGGAGTGCAAACGTCTGAA |
| U523 | TTAGAGTCGATGGGAAGACC |
| U549 | TGCTTTGTCTGTCATGTGAC |
| U550 | GAGACCGCAAAGGATTGTGA |
| U551 | GGATTCAGAGTGTCAGTGG |
| U606 | GATCTTTTCTACGGGGTCTG |
| U533 | CCTCAAGAGATCAAAGGGAA |
| U534 | GCAGGTTAGCAGTCTATAATG |
| U535 | GCAGTTACGTAGACTGCTGA |
| U536 | GTCCTTGGCGCTCTATAGAT |
| U537 | TGCCCAGGTACTGTATGCAG |
| U538 | CCCCTACTAGATTAGTTATCC |
| U539 | GAGTAGGCAGTAAAATTCTAAG |
| U540 | AATTACAATAACCGACCTTCC |
| U541 | TAGTAGTCGGTCTAGGAATC |
| U542 | CAAAGCTATAGAGACAGGCC |
| U553 | AACGGGCAGTGACATGGATG |
| U554 | GTAGGTTAGTGGTAGCTTCG |
| U556 | CTTGTCTTAGCCAGTTTCGG |
| U557 | GCTAACTAGCAGGTAAGTCC |
| U602 | GGCAATTTCGATGATGCAGC |
| U603 | TGCTGCCATAACCATGAGTG |
| U543 | CCCTAACCCTAACTTTTCCC |
| U544 | CCGTGTTATAACCCAACGGA |
| U545 | CATTGCTGGAAACAACGCTC |
| U546 | TGGCAACTGGGTCCTTTTC |
| U547 | CTTGGGGAAAGGATCAGAGC |
| U548 | TTGTTCTTGTCTCAAGGCGG |
| U552 | CCACATCCTGGAAGTGATAC |
| U558 | CGAACAATGTCGGATGGAAG |
| U559 | CAGTGAGTGTCTGCCTGAAT |
| U600 | TGGGGAGAAGTACCAAAAGG |
| U601 | CTAGATAGGGAAGTGACACG |
| U604 | GCCTGGACGACTAAACCAAA |
| U605 | CAATTTTCCAACGTCCGTGC |
| U611 | CTCTCAAGCAAGCCATCAAC |
| U612 | GGAGAGACTCACGATCTTGG |
| U613 | TGCCTTTAGAATATTCCTCCTCG |
| U614 | GTCTATCAAATCGCTCAAGCAAG |
| U615 | ATCTGGAGCTAATGCATCATCAC |
| U616 | ATGGGGTTTCGTTGTTTAATGAG |
| U617 | TATGACGATACAGGAAGTGCAAC |
| U618 | CTTCCAGTCAAAGTCATTCGATG |
| U619 | GATCCTGATCTATCGACACCATC |
| U620 | AAAACGCATTGAGATATCATGGG |
| U621 | GTGAAGTATCTGGACTCTCAAGG |
| U622 | GTTCCTGAGCATCAATCAATCTG |

Table S5

**Table S5.** Five transcription factors which do not have an essential gene function

| **Mutant** | **Gene ID** | **MIPS Functional annotation** | **PHI-base accesion number** | **Gene name** |
| --- | --- | --- | --- | --- |
| DAF139 | FGSG_00144 | related to ARG81 - transcription factor involved in arginine metabolism | PHI:1991 | GzZC306 |
| DAF140 | FGSG_16054 | hypothetical protein; transcription factor | PHI:1747; | GzZC062 |
|  | FGSG_16056 | hypothetical protein; transcription factor | PHI:1774 | GzZC089 |
| DAF141 | FGSG_06448 | conserved hypothetical protein | PHI:1714 | GzZC029 |
|  | FGSG_17682 | conserved hypothetical protein | PHI:1814 | GzZC129 |

# REFERENCES for Additonal File 1

1. Antoniw J, Beacham AM, Baldwin TK, Urban M, Rudd JJ, Hammond-Kosack KE: **OmniMapFree: a unified tool to visualise and explore sequenced genomes**. *BMC Bioinformatics* 2011, **12**:447.

2. Baldwin TK, Gaffoor I, Antoniw J, Andries C, Guenther J, Urban M, Hallen-Adams HE, Pitkin J, Hammond-Kosack KE, Trail F: **A partial chromosomal deletion caused by random plasmid integration resulted in a reduced virulence phenotype in *Fusarium graminearum***. *Molecular plant-microbe interactions : MPMI* 2010, **23**(8):1083-1096.

3. Lee SH, Kim HK, Hong SY, Lee YW, Yun SH: **A large genomic deletion in *Gibberella zeae* causes a defect in the production of two polyketides but not in sexual development or virulence**. *Plant Pathology J* 2006, **22**(3):215-221.

4. Gale LR, Bryant JD, Calvo S, Giese H, Katan T, O'Donnell K, Suga H, Taga M, Usgaard TR, Ward TJ *et al*: **Chromosome complement of the fungal plant pathogen *Fusarium graminearum* based on genetic and physical mapping and cytological observations**. *Genetics* 2005, **171**(3):985-1001.
